# Lipid stress evolved, microbiome‐based probiotics reduce lipid uptake in mice

**DOI:** 10.1002/btm2.70122

**Published:** 2026-02-01

**Authors:** Abhinav P. Acharya, Matthew A. Borrelli, Michael J. Jurczak, Jonathan Krakoff, Steven R. Little

**Affiliations:** ^1^ Department of Biomedical Engineering Case Western Reserve University Cleveland Ohio USA; ^2^ Department of Chemical Engineering University of Pittsburgh Pittsburgh Pennsylvania USA; ^3^ Division of Endocrinology and Metabolism, Department of Medicine University of Pittsburgh Pittsburgh Pennsylvania USA; ^4^ Center for Metabolism and Mitochondrial Medicine University of Pittsburgh Pittsburgh Pennsylvania USA; ^5^ Phoenix Epidemiology and Clinical Research Branch, National Institute of Diabetes and Digestive and Kidney Diseases National Institutes of Health Phoenix Arizona USA; ^6^ Department of Bioengineering University of Pittsburgh Pittsburgh Pennsylvania USA; ^7^ Department of Clinical and Translational Science University of Pittsburgh Pittsburgh Pennsylvania USA; ^8^ McGowan Institute for Regenerative Medicine University of Pittsburgh Pittsburgh Pennsylvania USA; ^9^ Department of Immunology University of Pittsburgh Pittsburgh Pennsylvania USA; ^10^ Department of Pharmaceutical Sciences University of Pittsburgh Pittsburgh Pennsylvania USA; ^11^ Department of Ophthalmology University of Pittsburgh Pittsburgh Pennsylvania USA

**Keywords:** evolution, lipid transport, probiotics, weight loss

## Abstract

Controlling the molecular transport of nutrients through the gut is an attractive strategy to modulate host metabolism. Herein, a technique of stress‐based evolution of an individual's own microbiota to enhance lipid metabolism is presented, which is based on sequential culture of these bacteria in higher concentrations of lipids. Using this technique, a probiotic formulation of bacterial colonies that exhibit increased lipid metabolism was generated from oral microbiota samples from mice, canine, and human sources. Mice fed a high‐fat diet (HFD) and administered lipid stress evolved (LSE) probiotics excreted increased lipids in stool and reduced triglyceride transport into the blood by three‐fold till 3 h post‐oral gavage of soybean oil, as compared to controls. In addition, these enhanced probiotics prevented weight gain in mice fed a HFD five‐fold better than controls and induced weight loss in mice with diet change three‐fold faster than diet change alone. In these mice, there was a marked change in appearance with a more healthy, less oily coat. Controlled metabolic cage experiments demonstrated that the total movement, food intake, and water intake were not significantly different between mice receiving LSE probiotic versus a control probiotic formulation, suggesting that important health measures are unchanged with LSE probiotic administration. Overall, this facile stress‐based culture technique can be utilized to modulate bacterial metabolism and applied to different industrial processes of probiotic generation and to affect different disease outcomes such as obesity.

**Translational Impact Statements**

Stress‐based evolution of an individual's own microbiota, achieved by sequential culture in elevated lipid concentrations, yields lipid stress evolved (LSE) probiotic formulations from mouse, canine, and human oral samples. In high‐fat diet mice, LSE probiotics increased fecal lipid excretion, reduced triglyceride transport into blood after oral lipid challenge, prevented weight gain fivefold better than controls, and accelerated diet‐induced weight loss threefold without changing activity or food intake. This simple, scalable method enables personalized or industrial probiotic development to modulate gut lipid transport and represents a promising, non‐invasive therapeutic strategy for obesity and related metabolic disorders.

## 1 INTRODUCTION

Obesity has become a global epidemic with more than 2 billion people now considered as overweight.^1^ Estimates suggest that within the U.S. general population, 73%–80% of male and 54%–60% of females are overweight or obese.^2^, ^3^ Obesity contributes to the development of numerous chronic disorders, including (but not limited to) cardiovascular disorders, diabetes, and cancer.^4^, ^5^, ^6^ In turn, the consequence of these disorders is increased risk of premature death and severely reduced overall quality of life. Importantly, over the last 15 years, the rate of obese individuals is increasing at an alarming rate.^7^, ^8^ Consequently, obesity incurs significant costs to the healthcare system that are continuing to rise. The annual medical expenditures have increased by more than $10 billion since the year 2000, for instance, including billions spent on obesity and overweight‐related morbidities, including $1.66 billion on diabetes care alone.^9^ Recently, glucagon‐like peptide 1 receptor agonists (GLP1Ra), or combination drugs, have shown considerable efficacy toward combating the obesity epidemic. However, weight loss maintenance following GLP1Ra treatment remains an unaddressed challenge. Lifestyle modifications commonly employed to maintain weight loss through dietary changes and/or changes to physical activity struggle with poor patient adherence/compliance, often resulting in refractory weight gain.^10^, ^11^ To prevent refractory weight gain, effectively combat overweight induced disorders, and to reduce the healthcare costs, there is an urgent need to develop innovative weight maintenance technologies with greater potential for patient compliance.

Regulation of intestinal dietary nutrient absorption or transport, including fat, amino acids, sugars, and minerals is critical for weight management. Some prescription drugs or techniques give the sensation of fullness or feeling less hungry (e.g., phentermine, the new GLP1 receptor agonists, or bariatric surgery); while other approaches (i.e., orlistat, a pancreatic lipase inhibitor) aim to prevent dietary fat absorption into the bloodstream to manage patient weight. However, these prescription drugs and surgeries produce undesirable side effects and are not as widely utilized as would be required to resolve the epidemic.^12^, ^13^, ^14^, ^15^, ^16^ Consequently, there is an increasing focus to treat obesity as a chronic condition and continue treatment after weight loss.^17^ Some of the GLP1‐based drugs also have potential to cause significant side effects, including gastrointestinal intolerance, reports of acute kidney injury, increased risk of gallbladder stones, increased heart rate, and injection site reactions, among others.^18^ Further, there may be greater than usual impact on fat‐free mass loss in particular on skeletal muscle.^19^ These drugs may also lead to reduction in cardiomyocyte size and cardiac mass.^20^ Despite their efficacy, widespread adoption is also limited due to a number of socioeconomic factors including employment status and insurance coverage.^21^ A 1 month supply of semaglutide, for instance, costs over $2000 for uninsured individuals,^22^ but insurance coverage is largely restricted to individuals whose BMI is >30 (or >27 with a comorbidity).^23^ Because obesity is overexpressed among individuals with a lower socioeconomic background,^24^, ^25^, ^26^ these therapeutics can often be unaffordable to their targeted population in United States.^27^ Taken together, there is a significant need to develop therapies that have low side effect profiles and high potential for compliance in order to achieve viable weight maintenance programs.

In contrast to GLP1Ra drugs, it may be viable to envision strategies to impact obesity that take advantage of the body's own natural mechanisms of intestinal absorption. Interestingly, in the intestines, the type and quantity of microbiota are known to depend on the type of diet^28^, ^29^ and at the same time, can modulate the absorption of nutrients (fat) through the intestines.^30^ It is reasonable to believe that engineering a patient's own microbiota could serve to limit fat absorption. Yet, current probiotic approaches are not effective in reducing weight because these do not directly address fat absorption/transportation.^31^, ^32^, ^33^

In this study, we demonstrate that stress‐based evolution of a patient's own gut bacteria can enhance lipid metabolism of the bacterial species. This enhanced metabolism could be utilized to decrease the transport of lipids through the intestines and thus reduce weight gain. Notably, we show that the resultant weight gain comes from a reduction in fat mass with no reduction in muscle mass. Importantly, this facile technology of stress‐based modification of the probiotics could potentially be integrated in probiotics generation in the industry for modulating different metabolic pathways and applied to different types of microbes for modulating disease outcomes.

## 2 RESULTS

### 2.1 Soybean lipid stress enhances lipase activity in commensal bacteria

**FIGURE 1:**
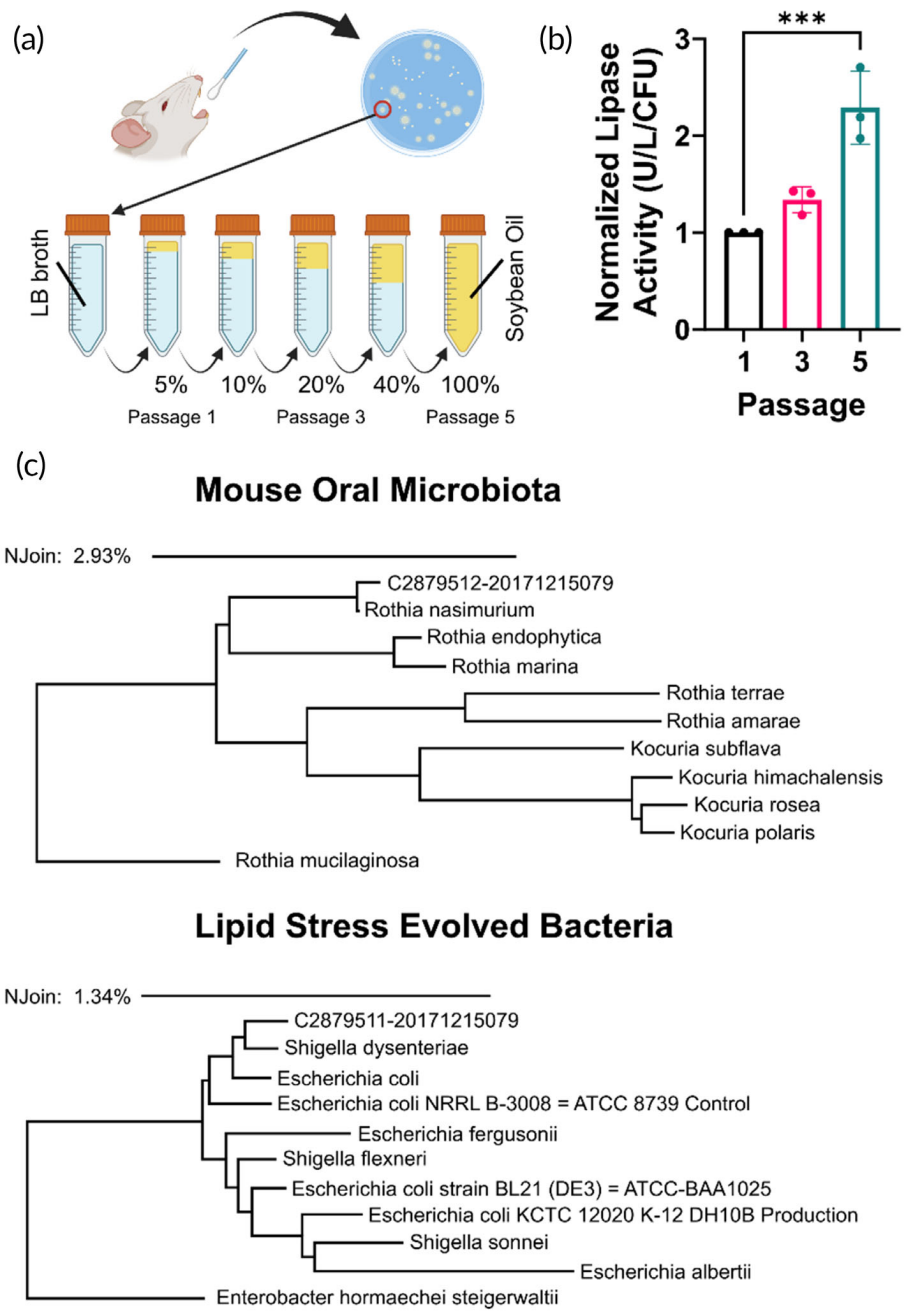
Directed evolution of commensal bacteria produces lipase‐secreting phenotype. (a) Directed evolution scheme—commensal oral microbes are plated on spirit blue agar and lipase‐secreting colonies are selected for expansion and evolution. In series passages with escalating lipid (soybean oil) content progress until a final incubation in 100% soybean oil. (b) Lipase activity from culture supernatants increases with increasing passages. (c) 16S RNA seek data show the lipid stress evolved bacteria are *Escherichia coli* /Shigella strain despite starting from commensal, lipase secreting Rothia organisms. Data represents mean ± SD for *N* = 3 colonies. One‐way ANOVA was performed with Dunnet's multiple comparisons correction. ***p < 0.001. CFU, colony forming units; LB, lysogeny broth.

To determine if lipid stressors can modulate the lipase function of commensal bacteria, saliva of C57BL/6j mice was isolated. These isolated bacteria were then cultured on a spirit blue agar plate with 0.3% Tween 80 as lipid source. The bacteria that grew on the plates and exhibited a white or clear halo (indicating lipase secretion) were selected for lipid stress evolution. This involves the subsequent passaging of bacteria through culture media with a lipid content (5%, 10%, 20%, 40%, and 100%) (Figure 1a). The lipase secretion was tracked through the evolution process, and it was observed that the lipase activity of the bacteria cultured in 100% Tween 80 solution was three‐fold higher than freshly isolated commensal bacteria (Figure 1b). The bacteria that survived the 100% lipid culture and grew on spirit blue agar were isolated and subjected to 16sRNA sequencing to determine the nearest neighboring microbial strain (Figure 1c). Notably, our bacterial culturing process selected for only facultative aerobic bacteria. It was determined that, for mice, this process selected for an *Escherichia coli*/Shigella strain (lipid stress evolved [LSE] bacteria) from a starting, lipase secreting, commensal, population of rothia organisms. This lipid metabolic stress evolution process was also applied to oral bacterial samples from canine and human sources and analyzed via 16sRNA sequencing, the results of which can be found in Figure S1. Similar to the samples derived from mice, the lipid evolution process selects for an *E. coli*/Shigella strain from a commensal oral microbiota starting point (Staphylococcus and Streptococcus; canine and human, respectively). Because this lipid stress evolution process yields similar organisms irrespective of the starting organism sample, it is reasonable to infer that the LSE strains carry similar mutations. Additionally, the LSE bacteria are almost exclusively Gram‐negative organisms, suggesting that the Gram‐positive organisms that predominate in the normal oral microbiota are unable to evolve in response to lipid stress. Future work examining the effect of lipid stress evolution under anaerobic culture conditions or with exclusively Gram‐positive organisms will serve to understand its limitations.

### 2.2 Lipid stress evolved bacteria exhibit increased metabolism of palmitic acid

**FIGURE 2:**
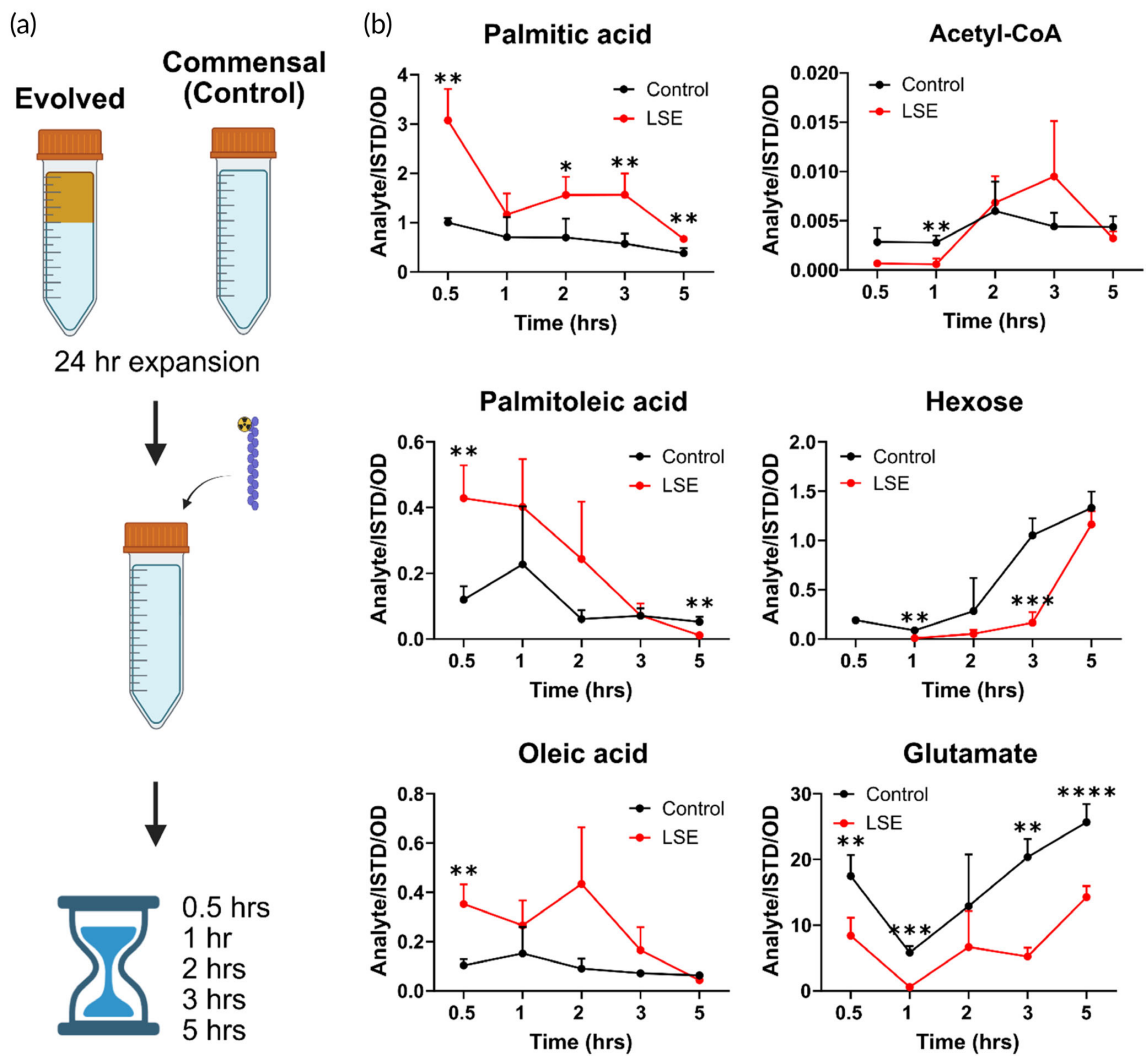
Metabolic study shows altered metabolites in lipid stress evolved (LSE) bacteria. (a) Study scheme—24 h expansion of frozen LSE bacteria or commensal (control) bacteria stock in 50% lipid and lysogeny broth (LB) broth, respectively. Next, the microbes are harvested, washed, and transferred to LB broth supplemented with 100 μM of C13 labeled palmitic acid, and sampling occurs at 0.5, 1, 2, 3, and 5 h following inoculation. (b) Quantification of key metabolites involved in fatty acid metabolism shows significant alterations in tracer uptake and reduced glucose metabolizing activity in LSE bacteria. Data represents mean ± SD for *N* = 6 cultures. Two‐way ANOVA was performed with Šídák's multiple comparisons test. *p < 0.05, **p < 0.01, ***p < 0.001, ****p < 0.0001.

To test if the lipid stress modulates lipid metabolism, the LSE bacteria and its commensal origin were expanded and 100 μM of 13C‐labeled palmitic acid was added to the culture media (Figure 2a). Figure 2b details key metabolites associated with fatty acids metabolism. Palmitic acid was upregulated in LSE bacteria significantly higher than control for all the time intervals tested except at 1 h. This suggests that the LSE bacteria have higher capacity to internalize palmitic acid. This coincided with elevated palmitoleic acid and oleic acid profiles, relative to the commensal control. Both palmitoleic and oleic acid metabolites arise from the elongation of palmitic acid by desaturase enzymes,^1^, ^34^ which suggests LSE bacteria have expanded palmitic acid metabolism. Notably, the levels of acetyl‐CoA, hexose, and glutamate were not enhanced in the LSE bacteria as compared to the controls. This suggests that LSE bacteria are not only able to internalize higher levels of the extracellular lipids but also metabolize these lipids for their energetic needs.

### 2.3 Lipid‐stressed bacteria reduce lipid transport from the intestine to the bloodstream

**FIGURE 3:**
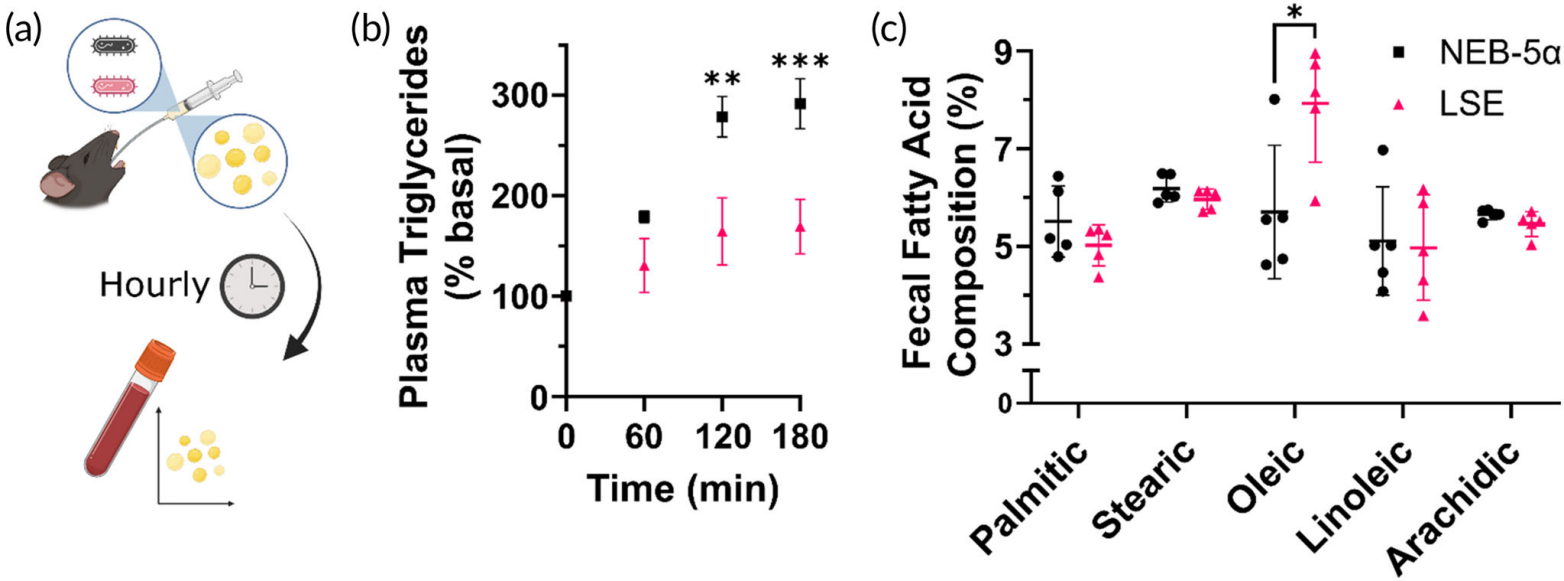
Lipid stress evolved (LSE) bacteria administration reduces fat uptake in real time. (a) Study scheme—mice are orally gavaged a probiotic formulation of NEB‐5α or LSE bacteria mixed with soybean oil. Blood samples are collected hourly following. (b) Plasma triglycerides following soybean oil and bacteria gavage are significantly depressed for LSE probiotic‐treated mice. (c) Fecal fatty acid profile shows greater excreted oleic acid following challenge. Data in (b) represents mean ± SD for *N* = 3 mice and data in (c) represents mean ± SD for *N* = 5 in vitro cultures. Two‐way ANOVA was performed with Šídák's multiple comparisons test for (b) and student's unpaired *t*‐test was performed for (c). *p < 0.05, **p < 0.01, ***p < 0.001.

To test if this enhanced lipid metabolism is also observed in vivo, C57BL/6j mice were orally gavaged with a probiotic formulation of LSE bacteria or a NEB‐5α control bacteria and soybean oil. NEB‐5α were used as controls here and throughout the project, since the DH5α *E. coli* bacterial strain is well characterized and does not have upregulated lipid metabolism.^2^, ^3^ Blood samples were collected following the gavage and quantified for triglyceride content (Figure 3a). It was observed that LSE probiotic reduced the triglyceride concentration in the plasma, as compared to the control bacteria (Figure 3b). Characterization of the long‐chain fatty acid content of fecal samples collected from mice following lipid and bacteria challenge reveals a greater excretion of oleic acid from mice receiving LSE probiotic (Figure 3c). Because fecal fatty acid profile correlates with the intestinal microbiota,^35^, ^36^ this finding demonstrates the in vitro lipid metabolism is conserved in vivo. Therefore, both in vitro and in vivo data suggest that LSE bacteria act to scavenge lipids from its environment resulting in less lipid transiting into the blood stream in the intestines. Future assessment of short‐chain fatty acids, which have been found to change in response to weight loss treatments,^4^ may further elucidate how in vivo fatty acid metabolism is altered by LSE administration.

### 2.4 Lipid stress evolved bacteria reduce weight gain from high‐fat diet and amplify weight loss from obese mice

**FIGURE 4:**
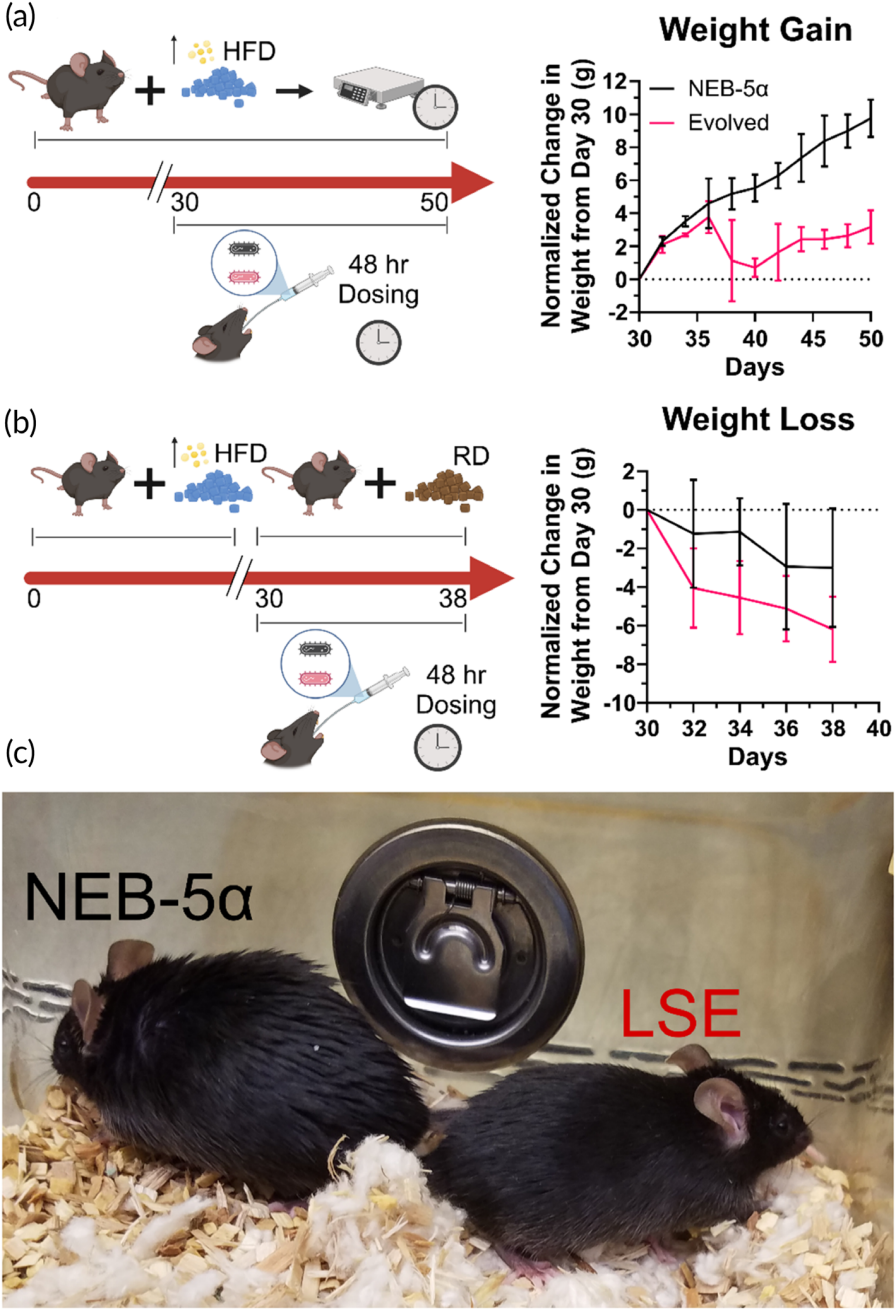
Lipid stress evolved (LSE) bacteria treatment reduces weight gain and enhances weight loss. (a) Weight gain study scheme—mice are fed a high‐fat diet (HFD) and weight is tracked for 50 days. On Day 30, and every 48 h following, mice are administered a probiotic formulation of LSE bacteria or a control NEB‐5α bacteria strain via oral gavage. Weight change, normalized to the start of treatment on Day 30, is reduced for mice administered LSE probiotic. (b) Weight loss study scheme—mice are fed a HFD for 30 days and then fed a regular diet (RD) for the remaining duration of the 38‐day study. On Day 30, and every 48 h following, mice are administered LSE probiotic or a control NEB‐5α probiotic via oral gavage. Weight loss, normalized to the start of treatment on Day 30, is accelerated for mice administered LSE probiotic. Figure S2 shows the weight change data without normalization for (a) and (b). (c) Representative image of NEB‐5α‐treated mice and LSE probiotic treated mice shows differences in mouse size and coat. Data represents mean ± SD for *N* = 3 mice.

Given that LSE bacteria have enhanced lipid metabolism and that these bacteria reduce lipid uptake in mice, the ability of these bacteria to modulate weight in high‐fat diet (HFD)‐induced obese mice was studied. Following 30 days of HFD to establish obesity, a probiotic formulation of LSE bacteria or NEB‐5α control were administered every 48 h and weight changes were recorded and plotted (Figures 4a and S2). Data suggest that the mice being administered LSE probiotic gained weight much slower than the mice that were fed NEB‐5α in the presence of HFD. Mice were also photographed at the end of the study. It was observed that the mice that received HFD and control bacteria had shinier fur and were larger than the mice that received lipid‐stressed bacteria (Figure 4c). Because shiny fur is associated with significant obesity in mice fed a HFD,^5^ this result qualitatively suggests LSE administration reduces lipid uptake by the host. Together, these data suggest that LSE probiotic could prevent lipid accumulation in the fur and tissue.

To determine how LSE bacteria may affect weight loss, mice were fed HFD for 30 days, then switched to a regular diet (RD) (12% kcal of fat), and administered a probiotic formulation of LSE bacteria or NEB‐5α control every 48 h. The change in weight over a period of 8 days was noted and plotted (Figures 4b and S2). These data suggest that LSE probiotics accelerated weight loss, as compared to the mice that were given NEB‐5α. These data further suggest that the lipid‐stressed bacteria may directly modulate the amount of fat absorbed in the gut. Therefore, the resulting deficit in dietary fat would require fat stores to be metabolized to meet the animal's needs, resulting in the observed weight loss.

### 2.5 Lipid stress evolved bacteria do not appreciably affect host metabolism

**FIGURE 5:**
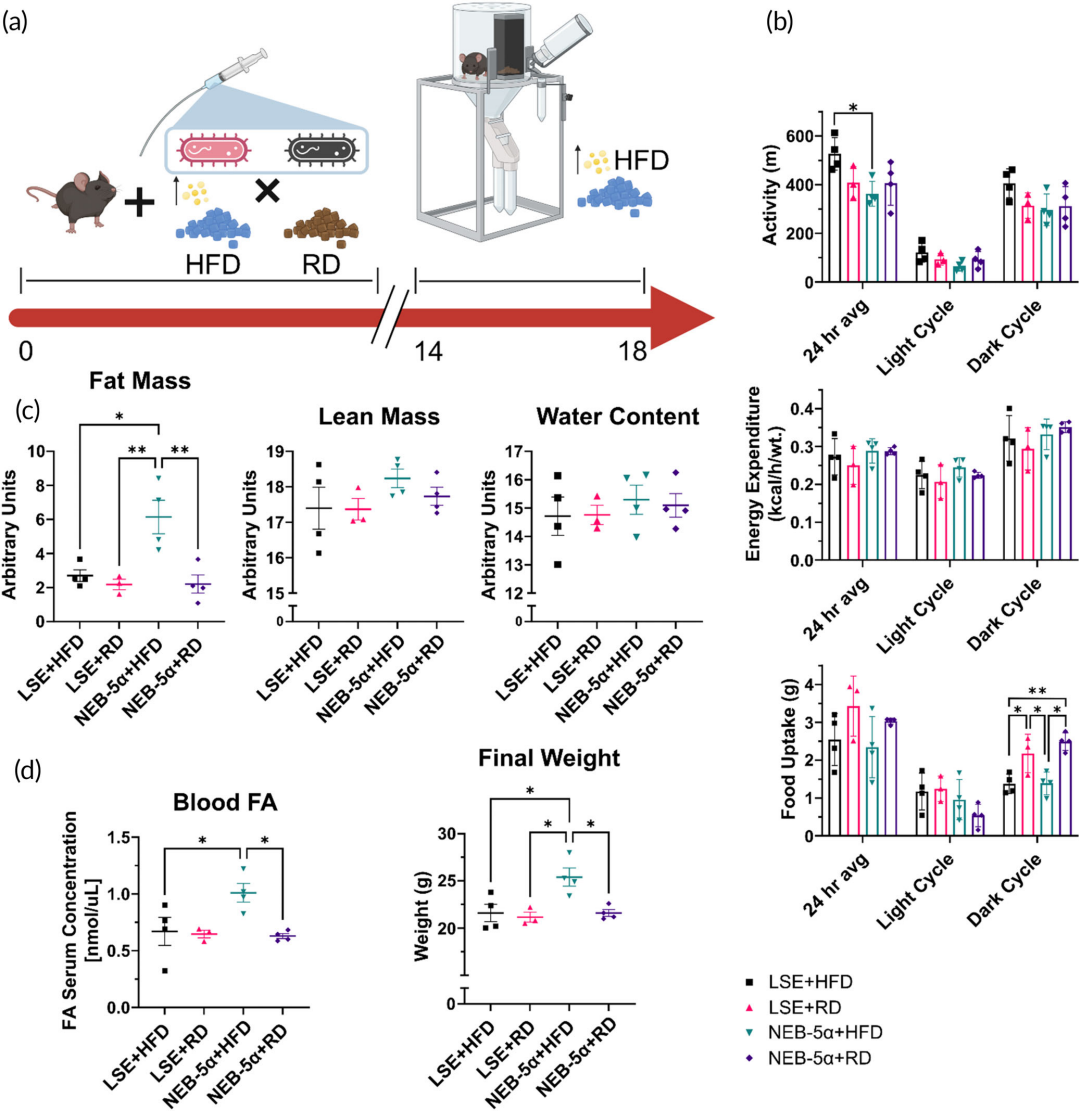
Metabolic cage experiments show lipid stress evolved (LSE) bacteria do not significantly alter the host metabolism. (a) Study scheme—mice were split into four groups receiving either a regular diet (RD) or high‐fat diet (HFD), and administered a probiotic formulation of either LSE bacteria or NEB‐5α control every 48 h for 14 days. On Day 14, the mice are transferred to a metabolic cage and given HFD. (b) Activity, food uptake, and calculated energy expenditure while in the metabolic cage were tracked. (c) Following the metabolic cage study, CT scans of the animals show significant reductions in fat mass for mice receiving HFD and LSE probiotic, relative to the NEB‐5α group. (d) Mice receiving HFD and LSE probiotic also have reduced fatty acid concentration in the serum and final weight, relative to the NEB‐5α group. Data represents mean ± SD for *N* = 3 to 4 mice. Statistical comparisons were performed using One‐way ANOVA with Dunnet's multiple comparisons correction. *p < 0.05; **p < 0.01.

We considered the possibility that LSE bacteria also modulate host metabolism. To determine if the energy expenditure (EE; a measure of metabolism of the host) is influenced by LSE bacteria, C57BL/6J mice were fed HFD or RD and were given a probiotic formulation of either LSE bacteria or NEB‐5α every alternate day for 14 days (Figure 5a). The mice were then transferred to metabolic cages and were kept on HFD for 96 h. Experiments were performed at a constant 30°C (thermoneutrality for mice) for the entire 96 h. Murine thermoneutrality was chosen for this experiment to ensure that the EE differences between the groups could not be attributed to differences in EE for maintaining body temperature. Once in the metabolic chamber, total movement, food intake, water intake, EE, and weight were recorded.

It was observed that the EE adjusted for lean mass were not significantly different from each other in LSE + HFD as compared to NEB‐5α + HFD; LSE + RD as compared to NEB‐5α + RD for the first 2 days (Figure 5b). Interestingly, the total movement between the lipid‐stressed + HFD and NEB‐5α + HFD was significantly different from each other for the first 24 h (Figure 5c). Notably, however, the total movement in the LSE group in the first 24 h was not significantly different from the controls of LSE + RD or NEB‐5α + RD. Taken together, these data suggest that lipid‐stressed bacteria might have improved the activity of mice to normal levels and are better than the control of NEB‐5α.

Moreover, the food uptake (Figure 5c) between the groups of mice getting the same diet (HFD or RD) was not significantly different, which strongly suggests important health measures are unchanged by LSE probiotic administration. Overall, these data suggest that the LSE probiotic did not modulate the metabolism of the host; appears to not influence the animals’ eating or drinking; and, potentially, induces normal movement in mice.

### 2.6 Fat mass, but not lean mass, is significantly lower in lipid stress evolved bacteria and HFD‐fed mice

Mice from the metabolic cage experiments described previously were sacrificed at its conclusion and Echo‐MRI DXA studies were performed. It was observed that there were no significant differences in fat mass between LSE + HFD, LSE + RD, and NEB‐5α + RD, and that these three groups were significantly lower than NEB‐5α + HFD (Figure 5d). These data strongly suggest that the difference in weight gain was due to fat mass loss in LSE + HFD, which supports the hypothesis that the lipid‐stressed bacteria are preventing the transport of lipids through the intestine and deposition in the body's tissues. Lean mass and water content between the groups was not significantly different from each other. This indicates that LSE probiotic did not cause any negative side effects to the host organism that may be expected from a probiotic. Moreover, the weight of the mice after the metabolic cage experiment was compared between the groups (Figure 5d). It was observed that the total weight of mice was significantly lower in the LSE + HFD as compared to the NEB‐5α + HFD group. Notably, this difference accounted for 2–4 g (or 10%–20% of the body weight), which has significant implications for preventing weight gain if this therapy were to be translated to larger animal models and humans.

## 3 DISCUSSION

The results presented in this study extend prior findings on microbial lipid metabolism and its role in host energy balance. The observed enhancement of lipase activity in commensal bacteria under lipid stress conditions echoes earlier work demonstrating that environmental stressors can induce metabolic shifts in gut microbiota. For instance, Turnbaugh et al. showed that the gut microbiome of obese individuals exhibits increased capacity for energy harvest from the diet, suggesting that microbial lipid metabolism is a modifiable factor in obesity pathogenesis.^37^ The directed evolution approach used here builds on this concept by selectively enriching for lipid‐metabolizing strains, offering a novel route to modulate intestinal lipid absorption. Although in this study soybean oil was utilized to enhance the lipid metabolism, other lipid molecules with complex structures may also be utilized to further enhance the repertoire of the bacteria to metabolize different types of lipids. In fact, modulating the lipid‐consuming ability of the microbiota was utilized here to reduce lipid uptake by the mice.

The in vivo reduction of plasma triglycerides and increased fecal oleic acid excretion following administration of LSE bacteria is consistent with studies that link microbial composition to lipid transport (Figure 3). For example, Velagapudi et al. demonstrated that gut microbiota can influence host lipid profiles by altering intestinal fatty acid uptake.^38^ The current findings suggest that LSE bacteria act as lipid scavengers, reducing systemic lipid exposure, a mechanism that may parallel the effects of orlistat, a lipase inhibitor, but with potentially fewer side effects.^39^ The LSE bacteria were enriched in soybean oil. Although soybean oil has approximately 60% polyunsaturated fatty acids (e.g., linoleic acid), 25% monounsaturated fatty acids (e.g., oleic acid), and 15% saturated fatty acids (e.g., palmitic acid),^40^ it was observed that only oleic acid was enhanced in the fecal samples of mice receiving LSE bacteria. This might indicate that the LSE procedure enhances the ability of the bacteria to metabolize monosaturated fatty acids more effectively than other fatty acids. This microbial intervention could represent a safer, more sustainable alternative to pharmacologic lipid absorption blockers. In addition to lipid uptake, other nutrients are also important to measure. This especially is true in case of glucose, which can be the primary reason for weight gain in humans. If the bacteria were glucose stressed to enhance their glucose metabolism, then this can provide a new avenue to further cause weight loss. One of the shortcomings of this study is that the microbiome of the mice post‐LSE bacteria feeding was not sequenced. Thus, it is not clear how long the effect of LSE lasts on the bacteria. This will be the focus of future studies.

The attenuation of weight gain and promotion of weight loss in HFD‐fed mice treated with LSE bacteria supports the growing body of evidence that gut microbiota can influence host adiposity. Studies such as those by Cani et al. and Everard et al. have shown that specific bacterial strains can modulate fat storage and inflammation, thereby impacting obesity outcomes.^41^, ^42^ The current data reinforce the therapeutic potential of microbiota‐based interventions, particularly those that target lipid metabolism directly. Importantly, the lack of lean mass loss in LSE‐treated mice distinguishes this approach from GLP‐1 receptor agonists (Figure 5), which have been associated with reductions in fat‐free mass, including skeletal muscle.^43^, ^44^, ^45^

The metabolic cage experiments further validate the safety profile of LSE bacteria (Figure 5), showing no significant changes in EE, food intake, or water consumption. These findings are in line with previous reports that probiotic administration does not adversely affect basal metabolic parameters.^46^ The normalization of movement in LSE‐treated mice may indicate a subtle improvement in overall health or energy levels, though further studies are needed to confirm this. The absence of metabolic disruption strengthens the case for LSE bacteria as a viable adjunct to obesity treatment. Finally, the significant reduction in fat mass without corresponding lean mass loss in LSE‐treated mice underscores the specificity of this intervention. Echo‐MRI data confirm that the weight differences are attributable to fat mass modulation, a finding that resonates with published literature^47^ which emphasize the importance of preserving lean mass during obesity treatment. The translational potential of this approach is further supported by the successful application of lipid‐stress evolution to human and canine microbiota, suggesting broad applicability across species. However, further experiments are needed to further ascertain the potential of this treatment before human use. Taken together, these results position LSE bacteria as a promising tool for weight maintenance and metabolic health, warranting further investigation in clinical settings.

The translation of this technology needs further work. For example, it will need to be demonstrated that this technology is reproducible, efficacious, and the mechanism is similar in multiple preclinical models to confirm that the LSE bacteria reliably reduce host lipid uptake. Moreover, comprehensive GLP safety and toxicology testing, covering dose‐range finding, biodistribution, persistence, horizontal gene transfer risk, and immunogenicity will need to be characterized. Also, early regulators will need to be engaged and Good Manufacturing Practice (GMP)‐grade manufacturing will need to be developed with clear release criteria for identity, potency, purity, genetic stability, and validated. Finally, phased clinical trials will need to be designed beginning with tightly controlled first‐in‐human studies that use sentinel dosing, objective lipid‐uptake biomarkers, microbiome sequencing, and long‐term follow‐up to evaluate safety, mechanism, and preliminary efficacy.

Overall, this study provides evidence that a lipid‐stress‐based method of directed bacterial evolution can be utilized to generate bacteria with increased lipid metabolism. These LSE bacteria can be effective in reducing weight gain in mice by reducing the transport of lipids through the intestinal tract. This technology, which leverages a lipid‐stress‐based evolution method to generate bacteria with enhanced lipid metabolism, holds promising applications in weight management and obesity treatment. This approach yields similar microbes in canine and human samples, indicating a high translation potential into future large animal and human studies. The ability to manipulate bacterial lipid metabolism could open new avenues for developing probiotic treatments that can aid in improving overall metabolic health. Combined with the public's familiarity with probiotic supplements, the affordability of probiotic supplements, and the reduced potential for serious side effects, a probiotic made from this fat metabolizing microbe may offer a novel and potentially effective means to combat obesity and/or facilitate weight maintenance following obesity treatment. Additionally, these lipid‐metabolizing microbes may also provide a novel approach to manage triglyceride levels, which is a critical risk factor of cardiovascular disease. Although this approach is highly promising, the selection of the bacterial strain is of paramount importance. For example, while evolving or expanding the bacteria, it is important to perform sequencing to ensure no pathogenic strain exists in the bacterial pool before administering to the host. Lastly, this facile strategy may be applied to stress‐based modulation of specific metabolic pathways, enabling the targeting of other metabolites that are overabundant in western society (i.e., carbohydrates).

## 4 METHODS

### 4.1 Isolation and generation of bacterial strains

Oral microbiota samples were obtained from mice, canines, and deidentified human donors, and expanded overnight in lysogeny broth (LB) media at 37°C (VWR 3500I Incubating Orbital Shaker, VWR International Radnor, PA) under aerobic conditions. A sample of the expanded commensal bacteria was frozen and retained for future studies. Samples of bacterial cultures were plated on spirit blue agar with 0.3% Tween 80 and incubated for 24–48 h at 37°C. Colonies that exhibited a white/clear halo around them, which indicates lipase secretion, were selected for metabolic stress‐based evolution following expansion in 2% Tween 80 (stock of Tween 80 = 1% Tween 80 in phosphate buffered saline (PBS)) and 98% LB broth.

To induce metabolic stress‐based evolution, these lipase secreting colonies were first expanded in a 50 mL culture of 5 vol.% soybean oil (lipid metabolite) and 95 vol.% LB media supplemented with 1 vol.% Tween 80 under aerobic conditions. After 24 h, bacterial cultures were centrifuged (Eppendorf 5810R 15 amp version, 1250 × g for 5 min at 20°C) and resuspended in 1 mL of LB media with 1 vol.% Tween 80. A sample of 100 μL of this bacterial suspension was taken forward for subsequent passaging in 10 vol.% lipid (balance 1 vol.% Tween 80 LB media). This process was repeated for 20% and 40% lipid cultures, until a final isolation step in 98 vol.% lipid and 2 vol.% PBS supplemented with 1 vol.% Tween 80. Bacteria surviving 24 h in this final lipid isolation were then plated on spirit blue agar and incubated for 24–48 h at 37°C. Colonies that exhibited a white/clear halo around them were selected for re‐expansion in 50% lipid (balance 1 vol.% Tween 80 LB media), which was flash frozen and kept as LSE bacteria stock, and for 16S RNA sequencing.

DH5α *E. coli* (NEB‐5α) bacterial strain was obtained from New England Biolabs (cat # C2987H, New England Biolabs, Ipswich, MA) and expanded per the suppliers’ specifications.

### 4.2 Metabolic study

Cultures of 50% lipid (balance 1 vol.% Tween 80 LB media) were inoculated with frozen stock of the LSE bacteria, and cultures of LB media were inoculated with frozen stock of commensal bacteria. Following a 24‐h expansion, bacterial cultures were centrifuged (1250 × g, 5 min, 20°C) and the pellet was washed 3 times with PBS. The bacterial pellets were then resuspended in LB media supplemented with 100 μM of C13 labeled palmitic acid (Cambridge Isotope Laboratories; Tewksbury, MA). Sacrificial sampling for analysis of fatty acids by liquid chromatography ‐ mass spectroscopy (LC–MS) was conducted after 0.5, 1, 2, 3, and 5 h of culturing at 37°C, and a sample was taken for optical density (OD) measurement (600 nm on SpectraMax M5; Molecular Devices; Sunnyvale, CA). Samples were submitted to the University of Pittsburgh Health Sciences Mass Spectrometry Metabolomics Core facility for analysis.

### 4.3 Analysis of fatty acids by LC–MS: Sample preparation

Metabolic quenching and polar metabolite pool extraction from 13C‐palmitate labeled bacterial cells was performed by adding ice cold 80% methanol (aqueous) at a ratio of 500 μL buffer per 1e6 cells. Deuterated (D3)‐creatinine and (D3)‐alanine, (D4)‐taurine, and (D3)‐lactate (Sigma‐Aldrich) were added to the sample lysates as an internal standard (STD) for a final concentration of 10 μM. Samples are scraped into Eppendorf tubes on ice, homogenized using a 25°C water bath sonicator and the supernatant was then cleared of protein by centrifugation at 16,000 x g. A 2 μL of cleared supernatant was subjected to online LC–MS analysis.

### 4.4 LC‐HRMS method

Analyses were performed by untargeted liquid chromatography ‐ high resolution mass spectroscopy (LC‐HRMS). Briefly, samples were injected via a Thermo Vanquish UHPLC and separated over a reversed phase Thermo HyperCarb porous graphite column (2.1 × 100 mm, 3 μm particle size) maintained at 55°C. For the 20‐min LC gradient, the mobile phase consisted of the following: solvent A (water/0.1% FA) and solvent B (ACN/0.1% FA). The gradient was the following: 0–1 min 1% B, increase to 15% B over 5 min, continue increasing to 98% B over 5 min, hold at 98% B for 5 min, re‐equilibrate at 1% B for 5 min. The Thermo IDX tribrid mass spectrometer was operated in both positive and negative ion mode, scanning in ddMS^6^ mode (2 μscans) from 70 to 800 *m*/*z* at 120,000 resolution with an Automatic Gain Control (AGC) target of 2e5 for full scan, 2e4 for ms2 scans using HCD fragmentation at stepped 15,35,50 collision energies. Source ionization setting was 3.0 and 2.4 kV spray voltage, respectively, for positive and negative mode. Source gas parameters were 35 sheath gas, 12 auxiliary gas at 320°C, and eight sweep gas. Calibration was performed prior to analysis using the PierceTM FlexMix Ion Calibration Solutions (Thermo Fisher Scientific). Peak identity was confirmed from an in‐house library with respect to accurate mass and retention time. Peak areas were manually extracted using Thermo QuanBrowser ver4.3, normalized to internal standards. Background natural 13C abundance of isotopologies were subtracted out using the Mass Isotopomer Multi‐Ordinate Spectral Analysis (MIMOSA) technique, before graphing using GraphPad PRISM 9.0.

### 4.5 Triglyceride uptake in mice

Oral microbiota obtained from C57BL/6j mice were subjected to lipid metabolic stress‐based evolution and the LSE bacteria were flash frozen and lyophilized yielding a probiotic formulation. These mice were then orally gavaged with 10^7^ colony forming units (CFU) of the LSE probiotic or a control probiotic (NEB‐5α) suspended in 50 μL of soybean oil. NEB‐5α were used as controls here and throughout the project because these bacteria are well characterized and do not have upregulated lipid metabolism.^48^, ^49^ Following gavage, blood samples were collected via tail vein bleed every hour for 3 h and a triglyceride assay (Sigma‐Aldrich; St. Louis, MO) was used to quantify the triglyceride content in the blood samples. Fecal samples were later collected and solvated in 50% aqueous acetonitrile at a concentration of 50 mg/mL. A total of 5 μg/mL deuterated internal standards: myristoleic acid‐d_27_, palmitic acid‐d_31_, linoleic acid‐d_11_, palmitoleic acid‐d_14_, oleic acid‐d_17_, arachidonic acid‐d_8_, stearic acid‐d_35_ (CDN Isotopes, Quebec, Canada) were added. Samples were homogenized using a FastPrep‐24 system (MP‐Bio), with Matrix D at 60 hz for 30 s, before being cleared of protein by centrifugation at 16,000 x g. A total of 600 μL cleared supernatants were dried to completion under N_2_ gas. Samples were reacted with 200 μL oxalyl chloride (200 mM in 2 M dichloromethane) at 65°C for 5 min and dried to completion under N_2_ gas. Free fatty acids were converted into FA‐PA derivatives by reacting with 150 μL 3‐picolylamine (1% in acetonitrile) for 5 min at room temp before being dried to completion in N_2_ gas. Samples are then resuspended in 500 μL ethanol. FA‐PA calibration curves of purified standards were prepared as above, serially diluted from 100 to 15 ng/mL. Samples were submitted to the University of Pittsburgh Health Sciences Mass Spectrometry Lipidomics Core facility for analysis.

### 4.6 LC–MS analysis

Derivatized samples were injected (5 μL) via a Thermo Vanquish UHPLC and separated over a reversed phase Phenomenex Luna 100 mm × 2.1 mm 3.0 μM particle C8 maintained at 55°C. For the 15‐min LC gradient, the mobile phase consisted of the following: solvent A (water/0.1% acetic acid) and solvent B acetonitrile (ACN/0.1% acetic acid). The gradient was as follows: 0–0.1 min 35% B, increase to 60% B over 7 min, continue increasing to 99% B over 0.1 min, hold at 100% B for 2 min, re‐equilibrate at 35% B for 4 min. The Thermo IDX tribrid mass spectrometer was operated in positive ion mode, scanning in data‐dependent MS (ddMS)^6^ mode (2 μscans) from 75 to 1000 *m*/*z* at 120,000 resolution with an AGC target of 2e5 for full scan, 2e4 for ms2 scans using High energy collisional dissociation (HCD) fragmentation at stepped 15, 35, 50 collision energies. Source ionization setting was 3.0 kV spray voltage, respectively, for positive mode. Source gas parameters were 45 sheath gas, 12 auxiliary gas at 320°C, and three sweep gas. Calibration was performed prior to analysis using the PierceTM FlexMix Ion Calibration Solutions (Thermo Fisher Scientific). Integrated peak areas were then extracted manually using Quan Browser (Thermo Fisher Xcalibur ver. 2.7). Flowing Atmosphere ‐ Presssure Afterglow (FA‐PA) are reported as an area ratio of FA to the internal standard and converted to absolute concentration via calibration curves.

### 4.7 Mouse models of weight change

A diet‐induced obesity (DIO) model of C57BL/6J, in which mice received a HFD (60% kcal of fat), was utilized. To evaluate the prevention of weight gain, age‐matched mice (6–8 weeks old) were fed HFD for 50 days, and on Day 30 they were administered either the LSE probiotic or NEB‐5α control via oral gavage (CFU = 10^7^/50 μL saline) every 48 h until the conclusion of the study. In a subsequent study to evaluate weight loss, age‐matched mice (6–8 weeks old) were fed HFD for 30 days and then switched to a RD (12% kcal of fat) until the conclusion of the study. On Day 30 probiotic administration began every 48 h as described for the prevention of weight gain study above. For both studies, mice were weighed every 48 h and qualitative indicators of animal obesity, such as size and coat sheen, were noted.

### 4.8 Metabolic cage experiments

Age‐matched mice (6–8 weeks old) were fed HFD or RD and were given either LSE probiotic or NEB‐5α every alternate day for 14 days yielding four experimental groups: LSE + HFD, LSE + RD, NEB‐5α + HFD, and NEB‐5α + RD. On Day 14, the mice were transferred to metabolic cages and were kept on HFD for 96 h. The metabolic cage was maintained at a constant 30°C the thermoneutrality point for mice and total movement, food and water intake, EE, and weight were recorded for 96 h. At the conclusion of the study, blood samples were obtained to analyze free fatty acid (Sigma‐Aldrich; St. Louis, MO). Mice were then sacrificed, and body composition (fat/lean mass) was measured by proton‐NMR using the Echo Magnetic Resonance Imaging (EchoMRI)‐100H platform (EchoMRI, Houston, TX).

## AUTHOR CONTRIBUTIONS

APA and SRL conceived of the idea, APA and MAB performed experiments and wrote the manuscript, MJJ performed metabolic cage experiments and performed data analyses, JK and all authors edited the manuscript.

## CONFLICT OF INTEREST STATEMENT

Abhinav P. Acharya and Steven R. Little have a patent on the technology described here—US20230210918A1.

## Supporting information


**Data S1.** Supporting Information.

## Data Availability

The data that support the findings of this study are available from the corresponding author upon reasonable request.
