# Supplementary material for: Lipid stress evolved, microbiome‐based probiotics reduce lipid uptake in mice
Source: Bioeng Transl Med. 2026 Feb 1;11(2):e70122. doi: 10.1002/btm2.70122 (PMC13093803; doi:10.1002/btm2.70122)
Supplement: Supplementary file 1 — Data S1. Supporting Information. [file BTM2-11-e70122-s001.docx]

**Supplementary Figures**

**Lipid-Stress-Evolved, Microbiome-Based Probiotics Reduce Lipid Uptake in Mice**

**Abhinav P. Acharya^1,#^, Matthew A. Borrelli^2,#^, Michael J. Jurczak^3, 4^, Jonathan Krakoff^5^, Steven R. Little^2, , 6, 7, 8, 9, 10, 11, *^**

1. Department of Biomedical Engineering, Case Western Reserve University, Cleveland, OH, 44106, USA.
2. Department of Chemical Engineering, University of Pittsburgh, 940 Benedum Hall, 3700 O'Hara Street, Pittsburgh, PA, 15213, USA.
3. Division of Endocrinology and Metabolism, Department of Medicine, University of Pittsburgh, Pittsburgh, PA, USA
4. Center for Metabolism and Mitochondrial Medicine, University of Pittsburgh, Pittsburgh, PA, USA.
5. Phoenix Epidemiology and Clinical Research Branch, National Institute of Diabetes and Digestive and Kidney Diseases, National Institutes of Health, Phoenix, Arizona, USA.
6. Department of Bioengineering, University of Pittsburgh, 302 Benedum Hall, 3700 O'Hara Street, Pittsburgh, PA, 15260, USA.
7. Department of Clinical and Translational Science, University of Pittsburgh, Forbes Tower, Suite 7057, Pittsburgh, PA, 15213, USA.
8. McGowan Institute for Regenerative Medicine, University of Pittsburgh, 450 Technology Drive, Suite 300, Pittsburgh, PA, 15219, USA.
9. Department of Immunology, University of Pittsburgh, 200 Lothrop Street, Pittsburgh, PA, 15213, USA.
10. Department of Pharmaceutical Sciences, University of Pittsburgh, 3501 Terrace Street, Pittsburgh, PA, 15213, USA.
11. Department of Ophthalmology, University of Pittsburgh, 203 Lothrop Street, Pittsburgh, PA, 15213, USA.

* Corresponding Author: [srlittle@pitt.edu](mailto:srlittle@pitt.edu).

# Equal Contribution

**Supplementary Figure 1 – Oral microbiota from different source organisms yields an E-coli/Shigella strain following lipid stress evolution**

Bacterial samples were plated on spirit blue agar to select for lipase secreting colonies in the commensal microbes and the final, lipid stress evolved organism.

**
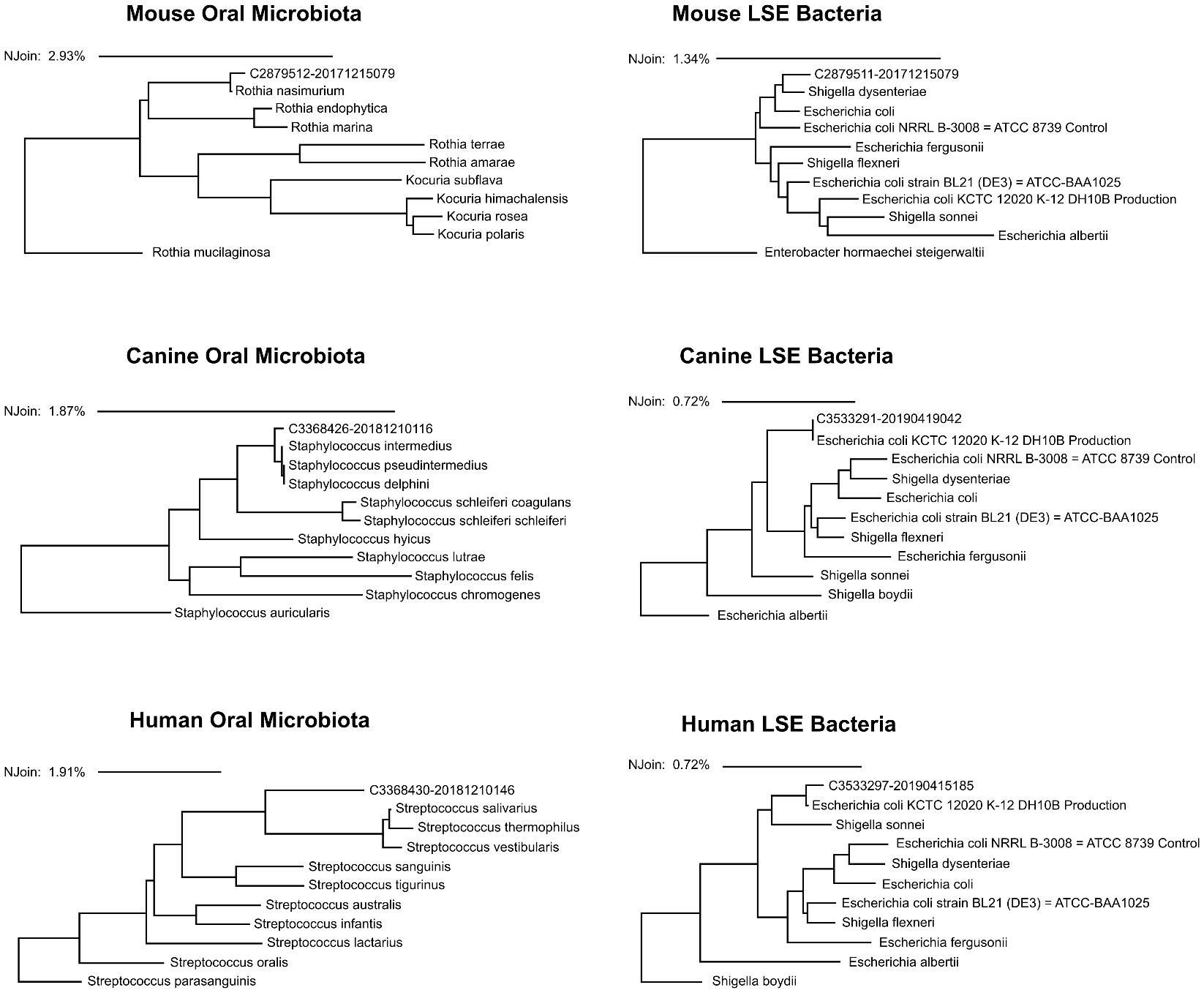
**

**
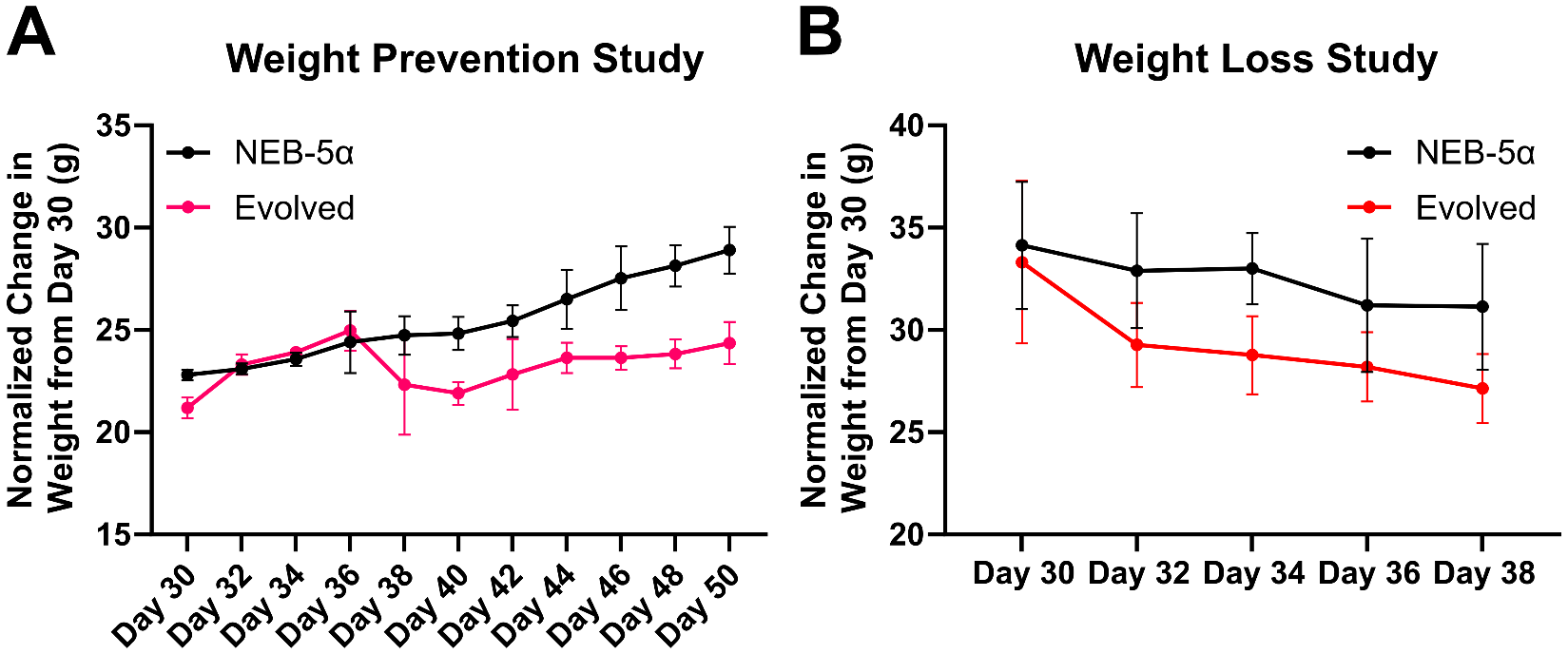
**

**Supplementary Figure 2 – Lipid stress evolved bacteria treatment reduces weight gain and enhances weight loss.**

(**A**) Weight change is reduced for Mice administered LSE probiotic in the presence of HFD.

(**B**) Weight loss is accelerated for Mice administered LSE probiotic, after change of the diet.
